# Forsythoside B attenuates memory impairment and neuroinflammation via inhibition on NF-κB signaling in Alzheimer’s disease

**DOI:** 10.1186/s12974-020-01967-2

**Published:** 2020-10-15

**Authors:** Fan’ge Kong, Xue Jiang, Ruochen Wang, Siyu Zhai, Yizhi Zhang, Di Wang

**Affiliations:** 1 School of Life Sciences, Jilin University, Changchun, 130012 China; 2 Department of Neurology, the Second Hospital of Jilin University, Jilin University, Changchun, 130041 China

**Keywords:** Forsythoside B, Alzheimer’s disease, Neuroinflammation, NF-κB

## Abstract

**Background:**

Neuroinflammation is a principal element in Alzheimer’s disease (AD) pathogenesis, so anti-inflammation may be a promising therapeutic strategy. Forsythoside B (FTS•B), a phenylethanoid glycoside isolated from *Forsythiae fructus*, has been reported to exert anti-inflammatory effects. However, no studies have reported whether the anti-inflammatory properties of FTS•B have a neuroprotective effect in AD. In the present study, these effects of FTS•B were investigated using amyloid precursor protein/presenilin 1 (APP/PS1) mice, BV-2 cells, and HT22 cells.

**Methods:**

APP/PS1 mice were administered FTS•B intragastrically for 36 days. Behavioral tests were then carried out to examine cognitive functions, including the Morris water maze, Y maze, and open field experiment. Immunohistochemistry was used to analyze the deposition of amyloid-beta (Aβ), the phosphorylation of tau protein, and the levels of 4-hydroxynonenal, glial fibrillary acidic protein, and ionized calcium-binding adapter molecule 1 in the hippocampus. Proteins that showed marked changes in levels related to neuroinflammation were identified using proteomics and verified using enzyme-linked immunosorbent assay and western blot. BV-2 and HT22 cells were also used to confirm the anti-neuroinflammatory effects of FTS•B.

**Results:**

In APP/PS1 mice, FTS•B counteracted cognitive decline, ameliorated the deposition of Aβ and the phosphorylation of tau protein, and attenuated the activation of microglia and astrocytes in the cortex and hippocampus. FTS•B affected vital signaling, particularly by decreasing the activation of JNK-interacting protein 3/C-Jun NH2-terminal kinase and suppressing WD-repeat and FYVE-domain-containing protein 1/toll-like receptor 3 (WDFY1/TLR3), further suppressing the activation of nuclear factor-κB (NF-κB) signaling. In BV-2 and HT22 cells, FTS•B prevented lipopolysaccharide-induced neuroinflammation and reduced the microglia-mediated neurotoxicity.

**Conclusions:**

FTS•B effectively counteracted cognitive decline by regulating neuroinflammation via NF-κB signaling in APP/PS1 mice, providing preliminary experimental evidence that FTS•B is a promising therapeutic agent in AD treatment.

## Background

Alzheimer’s disease (AD) is the main cause of dementia, which will affect 131.5 million people worldwide by 2050 [1]. AD patients are characterized by extracellular plaques of aggregated amyloid-beta (Aβ) and neurofibrillary tangles composed of hyperphosphorylated tau protein, and neuronal loss in their central nervous system. AD patients show clinical symptoms of memory loss, cognitive disorder, and neuropsychiatric symptoms, and require heavy economic cost on patient care and treatment [2]. However, the current treatment methods have problems of inefficiency, tolerance, and side-effects [3]. N-methyl-D-aspartate receptor antagonists only relieve dementia symptoms for a limited time and cannot stop or reverse AD progression [3]. Acetylcholinesterase inhibitors lose efficacy after chronic usage and have uncontrollable adverse effects [4]. Finally, long-term prophylactic use of non-steroidal anti-inflammatory drugs may reduce the risk of AD development, although the harmful side effects of these drugs limit their use [5].

Neuroinflammation is demonstrated to involve in AD pathogenesis by a large amount of evidence [6]. Neuroinflammation is characterized by reactive gliosis surrounding the amyloid plaques. The activation of chronic glial and the production of pro-inflammatory cytokine stimulate neurodegenerative, which are observed in AD patients and animal models [7]. Inflammation activated microglia and astrocytes produce tumor necrosis factor-alpha (TNF-α) and nitric oxide (NO) to exacerbate neuronal damage in several neurodegenerative diseases [8, 9]. Activated microglia and astrocytes produce interleukin (IL)-6 and IL-1β to increase the level of amyloid-beta precursor protein (APP) and promote Aβ deposition in AD mice models [10]. Nuclear factor-κB (NF-κB) is also activated in microglia and astrocytes and aggravates neurodegeneration in rat hippocampus [11, 12]. Therefore, the control of neuroinflammation may be a feasible therapeutic strategy in AD.

Natural products with anti-inflammatory actions may exert positive effects in patients with AD [13]. *Forsythiae Fructus*, the fruit of *Forsythia suspensa* (Thunb.) Vahl, has traditionally been used to treat inflammation and pyrexia in Korea, China, and Japan [14]. More than 30 phenylethanoid glycosides that exhibit anti-inflammatory, anti-bacterial, and anti-oxidant effects have been isolated from *Forsythiae Fructus* [15]. We have found that forsythoside B (FTS•B), which has the chemical structure C_34_H_44_O_19_ (Figure S1), exhibits better neuroprotection than other phenylethanoid glycosides of *Forsythiae Fructus* based on cell screening. FTS•B alleviates lipopolysaccharide (LPS)-induced acute lung injury by attenuating the infiltration of inflammatory cells and suppressing the activation of toll-like receptor (TLR)4/NF-κB signaling [16]. FTS•B suppresses the activation of NF-κB and ameliorates excessive inflammatory responses in sepsis patients [17]. FTS•B can rescue cardiac function by exerting an antioxidant effect that suppresses the inflammatory response [18]. However, FTS•B has not been addressed in the study of neuroprotection or modulation of neuroinflammation.

Therefore, we hypothesize FTS•B is anti-inflammatory and neuroprotective in AD mice. By using amyloid precursor protein/presenilin 1 (APP/PS1) AD mice model, we found FTS•B ameliorated AD-like behaviors, prevented histopathological alterations, improved biochemical indicators, and suppressed the inflammatory response by regulating the NF-κB signaling pathway. In BV-2 microglial cells and hippocampal HT22 cells, we found FTS•B prevented lipopolysaccharide-induced neuroinflammation and reduced the microglia-mediated neurotoxicity. We provide evidence that FTS•B could be used to treat neuroinflammation in AD.

## Methods

### Cell culture

BV-2 microglial cells (CL-0493; Procell Life Science & Technology Co. Ltd., Wuhan, China) and HT22 cells (337709; BeNa Culture Collection, Beijing, China) were cultured in Dulbecco’s modified Eagle’s medium (DMEM; Gibco, Grand Island, NY) supplemented with 10% fetal bovine serum (Procell Life Science & Technology Co. Ltd.), 1% 100 μg/mL streptomycin and 100 units/mL penicillin (Gibco, Grand Island, NY, USA). The cells were maintained at 37 °C in a humidified incubator containing 5% CO_2_ (Thermo Fisher Scientific Inc., Waltham, MA, USA).

### Cell viability and apoptosis assay

BV-2 cells seeded into 96-well plates were pretreated with 1 μM and 2.5 μM FTS•B (98%; Shanghai Yuanye Bio-Technology Co. Ltd., Shanghai, China) or phosphate-buffered saline (PBS) for 3 h. They were then co-incubated with 1 μg/mL LPS (Sigma-Aldrich, St. Louis, MO, USA) dissolved in DMEM for 24 h. Cell viability was analyzed using the 3-(4,5-dimethyl-2-thiazolyl) 2,5-diphenyl-2H-tetrazolium bromide (MTT; Sigma-Aldrich, USA) assay, as in our previous study [19].

In a separate experiment, BV-2 cells were pre-incubated with 1 μM and 2.5 μM FTS•B or PBS at 37 °C for 3 h. They were then co-incubated with 1 μg/mL LPS for 24 h and the culture media were collected. HT22 cells seeded into 12-well plates were then exposed to the collected medium for another 24 h. Cell viability and apoptosis of HT22 cells were analyzed using the MTT assay and annexin V/propidium iodide (AV/PI) staining, as in our previous study [20].

### Animal study

#### Acute toxicity test

Male C57 mice (8 weeks, 20-25 g) obtained from Liaoning Changsheng Biotechnology Co. Ltd. (Liaoning, China) were used to evaluate the acute toxicity of FTS•B. The mice were given 400 mg/kg FTS•B via intragastric administration (*n* = 8/group). After FTS•B treatment, they were observed continuously for the first 4 h to monitor mortality and abnormal behaviors and then observed intermittently over the next 24 h. Thereafter, they were observed occasionally for the remainder of the 14-day study period to monitor any delayed effects. The mice were sacrificed on the 14th day after drug administration. Histological sections were taken to examine any damage to the liver, spleen, kidney, and brain [21].

#### Experiments performed on AD mice and agent administration protocol

Twenty-four double-transgenic (APPswePSEN1dE9/Nju) male B6C3-Tg mice aged 8 months and weighing 40-45 g were purchased from Nanjing Biomedical Research Institute of Nanjing University, Jiangsu, China (SCXK (SU) 2015-0001). The mice had the following genotype: (Appswe) T, (Psen1) T, and are referred to here as APP/PS1 mice. Eight wild type (WT) male mice of the same age and weight were also purchased. Their genotype was (Appswe) W, (Psen1) W. The mice were maintained under standard conditions (temperature: 23 °C ± 1 °C, humidity: 40–60%) with a 12-h light/dark cycle and food available ad libitum.

After a week of adjustable feeding, the APP/PS1 mice were randomly divided into three groups (*n* = 8/group) and intragastrically administered either 10 mg/kg FTS•B, 40 mg/kg FTS•B, or 5 mL/kg normal saline (model group) once a day for 36 days. Eight WT mice were orally administered 5 mL/kg normal saline for 36 days (control group). After the last behavioral test, the mice were euthanized by injection with 150 mg/kg 1.5% pentobarbital. Serum and tissues, including the brain, liver, spleen, and kidney, were collected for biochemical and histopathological analysis. The drug administration process and behavioral tests are shown in Figure S2.

### Behavioral tests

#### Morris water maze test

Brain function related to spatial learning and memory was assessed in the AD mice using the Morris water maze (MT-200; Chengdu, China) and the Water Labyrinth Video Tracking Analysis System (S7200; Chengdu Techman Software Co. Ltd., Chengdu, China). A circular pool was filled with water containing milk at a temperature of 22 -24 °C. A platform was placed 1 cm below the water surface and the pool was used for both training and experiment. Starting on the 29th day, the mice were trained for 5 min/day over 4 days. On the 33rd day, they were placed in the same quadrant of the pool, and the escape latency of mice to locate the hidden platform was recorded, up to a maximum of 60 s [20].

#### Y maze test

The spontaneous alteration test was assessed in a Y maze with three symmetrical arms measuring 300 × 200 mm at a 120° angle. The mice’s movements were recorded using a video camera mounted above the maze. On the 34th day, the mice were placed onto the distal end of one arm and expected to find the food on the other end within 5 min, which was the maximum time allowed by the system. On the 35th day, the time taken to find the food and the movement locus of the mice were recorded using the video camera [22].

#### Open field experiment

The open-field experiment test was performed in a soundproof dark box (bottom area: 30 × 30 cm, central region: 15 × 15 cm) to observe the mice’s autonomous behaviors in the new environment. On the 36th day, the mice were placed in a fixed central location and allowed to explore the environment freely for 5 min. The moving track and latency time of the mice in the center and surrounding areas were recorded by the instrument. The box was cleaned thoroughly after each individual test to avoid residual odors and dirt interfering with the results of the next test [23].

### Biochemical criteria detection

BV-2 cells were pretreated with FTS•B at doses of 1 μM and 2.5 μM for 3 h. They were then co-exposed to 1 μg/mL LPS for another 24 h. The levels of IL-6 (cat. No. KT2163-A), TNF-α (KT2132-A), inducible nitric oxide synthase (iNO_S_; KT2454-A), NO (MM-0658 M1), and IL-1β (KT2040-A) in the supernatant were measured using enzyme-linked immunosorbent assay (ELISA) kits (Jiangsu Kete Biotechnology Co., Ltd, Jiangsu, China) according to the manufacturer’s instructions.

The brain obtained from the APP/PS1 mice were homogenized in ice-cold PBS, and the protein concentration was detected using the Pierce™ bicinchoninic acid (BCA) protein assay kit (23227; Thermo Fisher Scientific, USA). The levels of the following factors were detected in the serum and the brain using ELISA kits, according to the manufacturer’s instructions (Jiangsu Kete Biotechnology Co. Ltd, Jiangsu, China): TLR3 (cat. No. KT9375-A), interferon regulating factor 3 (IRF3; KT9374-A), phosphor (p)-IRF3 (MM-50143 M1), interferon-beta (IFN-β; KT2124-A), C-Jun NH2-terminal kinase (JNK; KT9182-A), JNK-interacting protein 3 (JIP3; KT9377-A), p-JNK (KT9372-A), APP (KT2824-A), p-APP (KT9378-A), Aβ (KT9256-A), TNF-α (KT2132-A), IL-1β (KT2040-A), IL-6 (KT2163-A), IL-8 (KT2123-A), and IL-12 (KT2105-A).

### Hematoxylin and eosin (H&E) staining, thioflavine S staining, and immunohistochemistry examination

As in our previous study [24], 4% paraformaldehyde-fixed liver, spleen, kidney, and brain tissues were dehydrated using alcohol, embedded in paraffin, and cut into 5-μm standard sections. Specimens were stained using H&E and assessed using an inverted microscope (CKX41; Olympus, Japan).

Thioflavine S staining was used to visualize fibrillar Aβ deposits. After rehydration using xylene and a graded series of ethanol solutions, the brain slices were incubated with 1% thioflavine S in the dark for 30 min. They were then differentiated in 70% ethanol and washed with distilled water. After fixing with 50% glycerin in PBS, slices were observed using a fluorescent microscope (magnification ×100; Olympus Corporation, Tokyo, Japan) [25].

The brain sections were prepared as in our previous study [26]. The sections were incubated with primary antibodies against Aβ (dilution ratio 1:1000; ab2539), p-tau (p-s396; dilution ratio 1:4000; ab109390), 4-hydroxynonenal (4-HNE; dilution ratio 1:200; ab46545; Abcam, Cambridge, MA, USA), glial fibrillary acidic protein (GFAP; dilution ratio 1:200; bs-0199R), and ionized calcium-binding adapter molecule 1 (Iba1; dilution ratio 1:200; bs-1363R; Bioss Inc., Beijing, China) at 4 °C overnight. The following day, the sections were washed in PBS, incubated with horseradish peroxidase (HRP)-conjugated anti-rabbit secondary antibody (sc-3836; Santa Cruz Biotechnology Inc., Dallas, TX, USA) at 25 °C for 1 h, and incubated with streptavidin-organism HRP complex (Shanghai BestBio Science, Shanghai, China) at 25 °C for another 1 h. Next, 5% diaminobenzidine tetrahydrochloride solution and hematoxylin were added to the slices to counterstain (25 °C for 5 min). Photomicrographs were obtained using an inverted fluorescent microscope (magnification ×40 and ×200; Olympus Corporation, Tokyo, Japan).

### Proteomics

#### Protein extraction and digestion

Samples were homogenized in radio immunoprecipitation assay (RIPA) buffer containing 1% protease inhibitor cocktail (Sigma-Aldrich, St. Louis, MO, USA) and 2% phenylmethanesulfonyl fluoride (PMSF; Sigma-Aldrich, St. Louis, MO, USA) on ice for 30 min. After centrifugation, a BCA protein assay kit was used to quantify protein concentrations in the supernatant. After acetone precipitation, resuspension of the protein for tryptic digest, and removal of sodium deoxycholate, peptide samples were obtained. These were then desalted using reversed-phase chromatography on a C_18_ column, dried, and resuspended in buffer containing 0.1% formic acid (FA) and 2% acetonitrile (ACN).

#### Liquid chromatography coupled to tandem mass spectrometry

One microgram of peptide was separated and analyzed using an Easy-nLC1200 nano-UPLC (Thermo Scientific, Waltham, MA) connected to a Q-Exactive mass spectrometer (Thermo Scientific, Waltham, MA). Separation was performed using a reversed-phase column (100 μm, ID × 15 cm, Reprosil-Pur 120 C18-AQ, 1.9 μm, Dr. Math). A non-linear gradient was established using phase A (0.1% FA and 2% ACN in water) and phase B (0.1% FA and 80% ACN in water) to elute the peptides, with a 120-min gradient at a 300-nL/min flow rate. The gradient started at 8% phase B and was increased to 35% over 92 min. Phase B was increased to 45% over the next 20 min, then augmented to 100% over 2 min and kept at 100% for 2 min. Data-dependent acquisition for MS1 was performed in the profile and positive modes using an Orbitrap analyzer at a resolution of 70,000 (200 m/z) and an m/z range of 350-1600. For MS2, the resolution was set to 17,500, with a dynamic first mass. The automatic gain control target for MS1 was set to 3.0 E^+6^, with a maximum injection time (IT) of 50 ms, while that for MS2 was set to 5.0 E^+4^, with a max IT of 100 ms. The top 20 most intense ions were fragmented using higher-energy collisional dissociation, with a normalized collision energy of 27% and an isolation window of 2 m/z. The dynamic exclusion time window was 30 s.

#### MaxQuant analysis and label-free quantification

Raw MS files generated on the mass spectrometer were processed using MaxQuant (version 1.5.6.0). The protein sequence database (Uniprot_organism_2016_09) was downloaded from Uniprot. This database was then searched against its reverse decoy using the MaxQuant software. The quantification type was label-free quantification matched for run and intensity-based absolute quantification. Trypsin was set as a specific enzyme with up to three missed cleavage sites. Oxidation (M) and acetylation (protein N-term) were considered as variable modifications, and the maximum number of modifications per peptide was three. Carbamidomethyl (C) was set as a fixed modification. The false discovery rate for both peptide and protein was less than 0.01. Only unique and razor peptides were used for quantification. All other parameters were reserved as defaults.

#### Bioinformatics and statistical analysis

The standardized quantitative results were analyzed to obtain the corresponding differentially expressed proteins. The experiment comprised two biological replicates, and the arithmetical mean of these was used to calculate the fold change. Fold changes in expression of > 1.5 or < 0.66 were defined as significant differences, and subsequent gene ontology, Kyoto Encyclopedia of Genes and Genomes pathway and protein interaction analyses were performed.

### Western blot

As in previous studies, western blot analysis was carried out [9, 24]. One part of the hippocampal tissue obtained from the experimental mice was lysed and homogenized using RIPA buffer containing 1% protease inhibitor cocktail and 2% PMSF. The total loading protein (40 μg) was quantified using the BCA protein assay kit, separated on 12% sodium dodecyl sulfate-polyacrylamide gel electrophoresis, and subsequently transferred to a polyvinylidene difluoride membrane (0.45 μm; Merck Millipore, Billerica, MA, USA). Next, 5% bovine serum albumin was used to block the membranes at 4 °C for 2 h. The membranes were subsequently incubated with the following primary antibodies at 4 °C overnight: JIP3 (dilution ratio 1:500; 147 kDa; bs-13648R), JNK (dilution ratio 1:1000; 48 kDa; bs-2592R), APP (dilution ratio 1:1000; 86 kDa; bs-0112R), inhibitor of NF-κB kinase alpha+beta (IKKα+β; dilution ratio 1:500; 85 kDa; bs-7557R), IRF3 (dilution ratio 1:1000; 47 kDa; bs-52116R), p-IRF3 (Ser396; dilution ratio 1:500; 47 kDa; bs-3195R), Iba1 (dilution ratio 1:500; 16 kDa; bs-1363R), and GFAP (dilution ratio 1:500; 48 kDa; bs-0199R; Bioss Inc., Beijing, China), p-JNK1 + 2 + 3 (p-Y185 + Y185 + Y223; dilution ratio 1:10000; 48 kDa; ab76572), p-IKK(α + β; p-S176 + S177; dilution ratio 1:500; 85 kDa; ab194528), p-APP (p-T743; dilution ratio 1:1000; 86 kDa; ab206297), ELKS (dilution ratio 1:5000; 128 kDa; ab180507), inhibitor of nuclear factor kappa-B alpha (IκBα; dilution ratio 1:10000; 35 kDa; ab32518), p-IκBα (p-S36; dilution ratio 1:10000; 35 kDa; ab133462), NF-κB p65 (dilution ratio 1:500; 65 kDa; ab7970), and p-P65NF-κB (p-S536; dilution ratio 1:10000; 65 kDa; ab76302), WD-repeat and FYVE-domain-containing protein 1 (WDFY1; dilution ratio 1:1000; 46 kDa; ab125329), TLR3 (dilution ratio 1:500; 108 kDa; ab13915), and the reference protein glyceraldehyde-3-phosphate dehydrogenase (GAPDH; dilution ratio 1:500; 36 kDa; ab181602; Abcam, Cambridge, MA, USA). After washing with TBST (500 mL t-butyldimethylsilyl chloride and 0.5 mL Tween-20), the membranes were incubated with HRP-conjugated secondary antibody (SH-0032; Bejing Dingguo Changsheng Biotechnology Co. Ltd., Beijing, China) at a dilution of 1:2000 at 4 °C for 4 h. An enhanced chemiluminescence kit (Merck Millipore, Billerica, MA) and imaging system (Bio Spectrum 600; UVP company, Upland, CA, USA) were used to visualize the bands. The ImageJ software (National Institutes of Health, Bethesda, MD, USA) was used to quantify the blots.

### Statistical analysis

All data are presented as mean ± standard error. One-way analysis of variance followed by post hoc multiple comparisons (Holm–Sidak test) was used to analyze differences and significance using the SPSS 16.0 software (SPSS Inc., Chicago, IL, USA). Statistical significance was defined at *p* values < 0.05.

## Results

### Assessment of acute toxicity trials

The acute toxicity test of FTS•B was carried out in C57 mice. All mice remained alive and showed normal behavior after oral administration of 400 mg/kg FTS•B. There were no obvious behavioral abnormalities between the FTS•B and control groups either 4 h or 14 days after FTS•B administration. There were no significant changes in the liver, spleen, kidney, or brain after FTS•B administration (Figure S3), indicating that FTS•B has low toxicity.

### FTS•B-ameliorated cognitive decline and pathological alterations in APP/PS1 mice

Thirty-six days after FTS•B administration, there were no significant effects on body weight (Table S1) and no changes in the liver, spleen, kidney, or brain of APP/PS1 mice (Figure S4), indicating that FTS•B can be used safely.

**Fig. 1:**
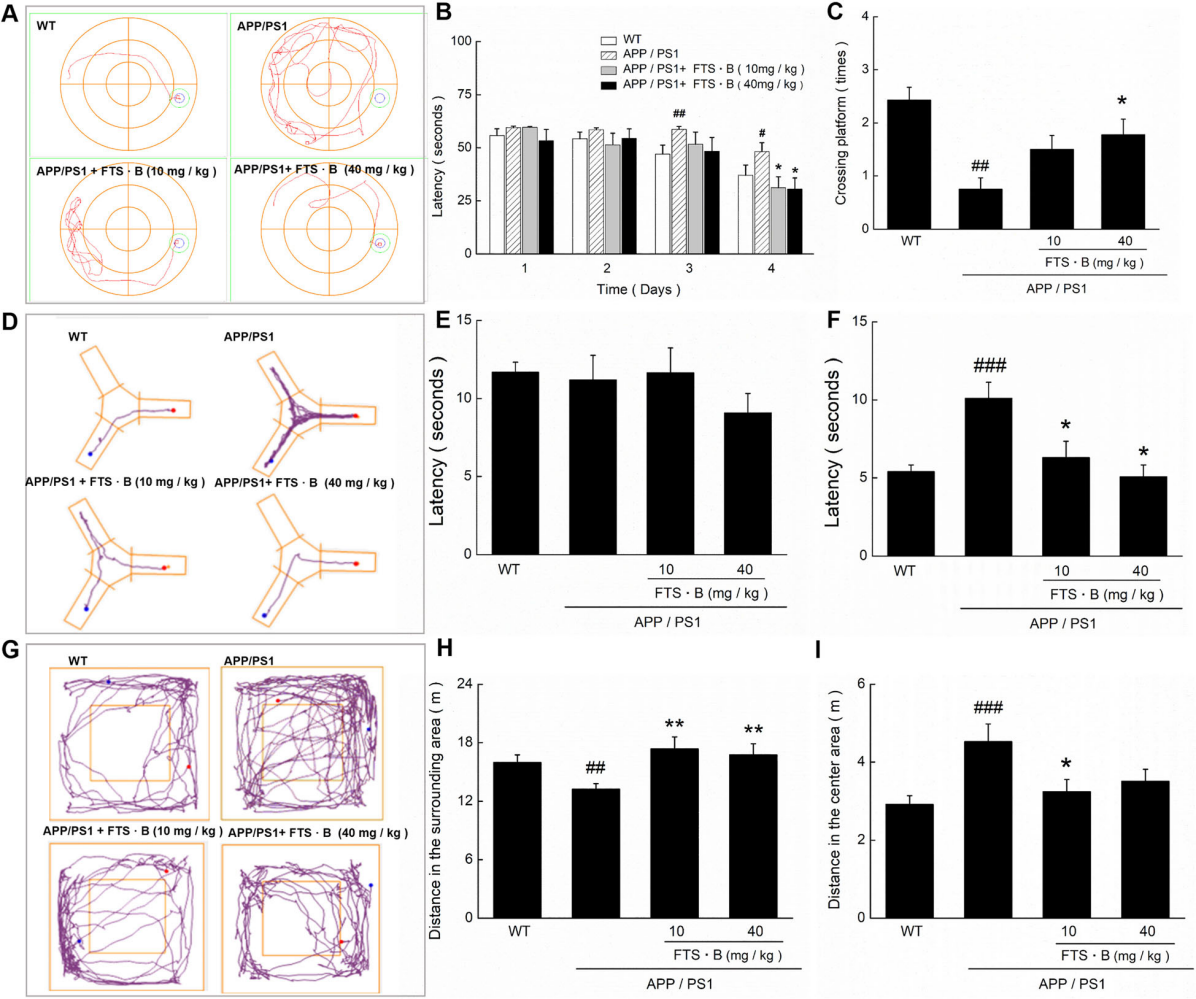
FTS•B counteracted cognitive decline in APP/PS1 mice. (**a**) In the Morris water maze test, the path tracings were automatically recorded on the testing day. FTS•B reduced (**b**) the escape latency on the 4 testing days in APP/PS1 mice, as well as (**c**) the times of crossing the platform on the testing day. (**d**) The path tracings were automatically recorded in the Y maze test. (**e**) FTS•B failed to influence training latency, but (**f**) strongly reduced the testing latency in the Y maze test. (**g**) The path tracings in the open field test were automatically recorded. (**h**) FTS•B increased the distance in the surrounding area and (**i**) reduced the distance in the center area of APP/PS1 mice in the open field test. Data are expressed as the mean ± standard error (*n* = 8). #*p* < 0.05, ##*p* < 0.01, and ###*p* < 0.001 vs. WT mice. *p < 0.05 and **p < 0.01 vs. APP/PS1 mice. FTS•B, forsythoside B; APP/PS1, amyloid precursor protein/presenilin 1; WT, wild type

The Morris water maze test has been widely used to evaluate learning and memory in animals [27]. Compared with vehicle-treated APP/PS1 mice, FTS•B-treated AD mice exhibited less chaotic movements (Fig. 1a) and shorter escape latencies (*p* < 0.05; Fig. 1b), and crossed the platform more times (*p* < 0.05; Fig. 1c).

The Y maze test is usually used to examine the short-term memory of mice [28]. In the Y maze test, the blue dot indicates the mice, the red dot indicates food, and the purple line indicates the movement tracks of the mouse. No visible differences in training time were noted among the experimental groups (*p* > 0.05; Fig. 1e). FTS•B suppressed the spontaneous alteration of behavior in APP/PS1 mice (Fig. 1d). In the formal test, mice administered FTS•B found the food in less time than vehicle-treated APP/PS1 mice (*p* < 0.05; Fig. 1f).

The mice’s autonomous exploration behaviors in a new environment were evaluated using the open-field experiment [29], wherein the blue dot signifies the initial location of the mice, the red dot indicates the terminal location of the mice, and the purple line denotes the movement tracks of the mouse. FTS•B-administered APP/PS1 mice showed significantly less aimless movement around the central field area (Fig. 1g) than vehicle-treated APP/PS1 mice, traveled a greater distance in the surrounding area (*p* < 0.01; Fig. 1h), and traveled less in the central area (*p* < 0.05; Fig. 1i). All of these data suggest that FTS•B strongly ameliorated cognitive decline in APP/PS1 mice.

**Fig. 2:**
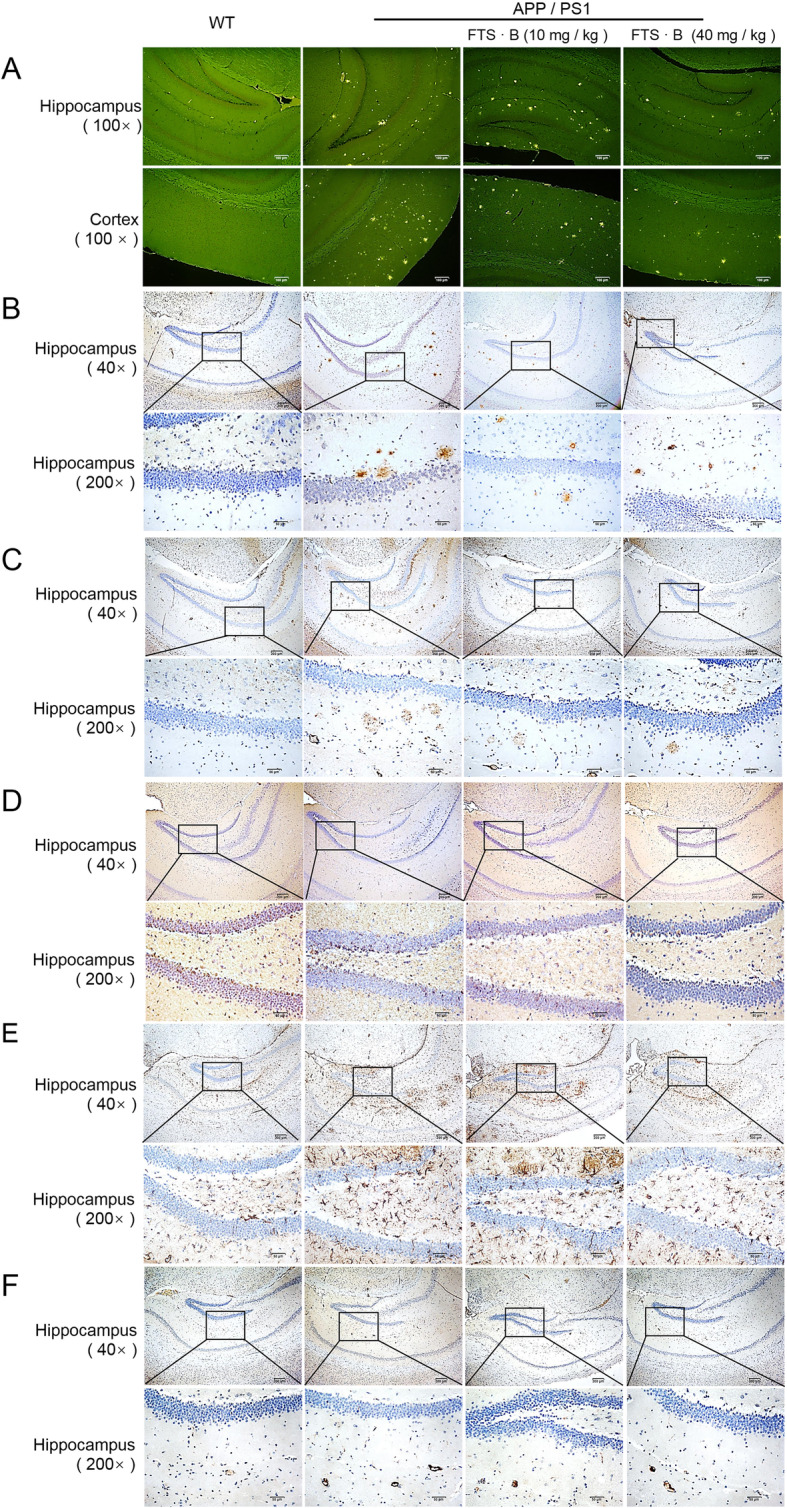
FTS•B ameliorated pathological alterations in the brains of APP/PS1 mice. FTS•B alleviated the deposition of Aβ in the brains of APP/PS1 mice, detected using (**a**) thioflavine S staining in the hippocampus and cortex (100×; scale bar: 100 μm; *n* = 3) and (**b**) immunohistochemistry in the hippocampus (40×; scale bar: 200 μm; 200×; scale bar: 50 μm; *n* = 3). FTS•B reduced the levels of (**c**) phospho-tau protein (*n* = 3), (**d**) 4-HNE (*n* = 3), and (**e**) GFAP (*n* = 3) in the hippocampus of APP/PS1 mice (40×; scale bar: 200 μm; 200×; scale bar: 50 μm). (**f**) FTS•B suppressed the enrichment of Iba1 around blood vessels in the hippocampus of APP/PS1 mice (*n* = 3; 40×; scale bar: 200 μm; 200×; scale bar: 50 μm). FTS•B, forsythoside B; APP/PS1, amyloid precursor protein/presenilin 1; 4-HNE, 4-hydroxynonenal; GFAP, glial fibrillary acidic protein; Iba1, ionized calcium-binding adapter molecule 1

Vehicle-treated APP/PS1 mice showed over-accumulation of fibrillary amyloid plaques of Aβ in the hippocampus and cerebral cortex, while these plaques were reduced in FTS•B-treated AD mice, as analyzed using thioflavine S staining (Fig. 2a). Immunohistochemistry showed that the area covered by Aβ positive plaques in the hippocampus of APP/PS1 mice was markedly larger than that in WT mice, and that this difference was ameliorated by FTS•B administration (Fig. 2b). As a microtubule-associated protein, tau stabilizes the neuronal cytoskeleton, and its hyperphosphorylation can lead to the formation of toxic neurofibrillary tangles in AD [30]. 4-HNE exists at higher concentrations in patients with AD and triggers Aβ aggregation [31]. In the present study, excessive aggregations of p-tau (Fig. 2c) and high expression of 4-HNE (Fig. 2d) were discovered in the hippocampus of vehicle-treated APP/PS1 mice compared to WT mice. All of these differences were strongly attenuated after FTS•B administration. Activated microglia and astrocytes around Aβ plaques are a representative characteristic of the AD brain and are conducive to the inflammatory process of brain damage [32]. Iba1 and GFAP are specific biomarkers of activated microglia and astrocytes, respectively [33], so immunohistochemistry of these proteins was used to assess the activation of microglia and astrocytes in the hippocampus. More GFAP-positive cells were noted in the hippocampus of APP/PS1 mice than in that of WT mice. This increase was markedly attenuated after FTS•B treatment (Fig. 2e). FTS•B also reduced the enrichment of Iba1 around blood vessels (Fig. 2f).

### FTS•B-regulated cytokine levels in serum and brain of APP/PS1 mice

**Fig. 3:**
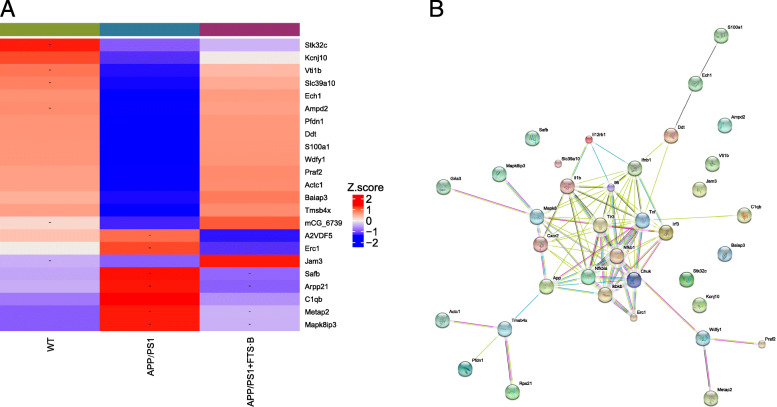
Label-free proteomics performed to evaluate the protein changes caused by FTS•B administration in the hippocampus of APP/PS1 mice. (**a**) The heatmap shows differences in the cellular protein profiles in the hippocampus between WT and APP/PS1 mice with or without FTS•B administration. (**b**) The protein interaction image shows that significantly up- or downregulated proteins may be closely related to inflammation. FTS•B, forsythoside B; APP/PS1, amyloid precursor protein/presenilin 1; WT, wild type

To analyze the specific effects of FTS•B in AD, proteomic analysis was performed on the hippocampus collected from each group. Among the detected proteins, 23 were markedly changed in AD (Table S2). A clustering heatmap of the significant proteins showed that FTS•B upregulated 16 proteins and downregulated 7 in the hippocampus of APP/PS1 mice (Fig. 3a). In the protein-protein interaction analysis, 116 interactions and 49 expected interactions were found (Fig. 3b).

Based on the screening data of proteomic analysis, the markedly changed cytokines were confirmed using ELISA. The over-expression of WDFY1, a potentiator of TLR3 signaling, can lead to elevated IRF3 phosphorylation and IFN-β gene expression [34]. FTS•B treatment, especially at 40 mg/kg, resulted in increases of 37.3% (*p* < 0.01), 12.5% (*p* < 0.05), and 76.1% (*p* < 0.001) in serum levels of TLR3, p-IRF3/IRF3, and IFN-β (Table 1), respectively, as well as 24.7% (*p* < 0.05), 150% (*p* < 0.001), and 52.6% (*p* < 0.05) increases in cerebral levels of TLR3, p-IRF3/IRF3, and IFN-β (Table 2). JNK is responsible for APP phosphorylation at Thr668 in differentiated neurons, which can generate Aβ peptide after two endoproteolytic cleavages [35]. Overproduction of JIP3, p-JNK/JNK, p-APP/APP, and Aβ was noted in the serum and brain lysate of APP/PS1 mice (*p* < 0.05) and was significantly reversed by FTS•B administration (*p* < 0.05; Tables 1 and 2). Aβ can activate neuroglia and stimulate the expression of pro-inflammatory chemokines such as TNF-α and ILs, perhaps leading to increased Aβ production and neuronal death [36, 37]. Compared with vehicle-treated APP/PS1 mice, FTS•B-treated specimens showed markedly lower levels of TNF-α (*p* < 0.01), IL-1β (*p* < 0.05), IL-6 (*p* < 0.01), IL-8 (*p* < 0.05), and IL-12 (*p* < 0.05) in the serum and brain lysate (Tables 1 and 2).

**Table 1:** The effects of FTS•B on cytokines levels in the serum of APP/PS1 mice

	WT	APP/PS1		
			FTS•B (mg/kg)	
			10	40
TLR3 (ng/mL)	29.6 ± 1.6	21.7 ± 1.9##	27.3 ± 1.7*	29.8 ± 1.2**
p-IRF3/IRF3	1.2 ± 0.1	0.8 ± 0.0##	0.9 ± 0.1*	0.9 ± 0.0*
IFN-β (pg/mL)	95.4 ± 7.0	51.5 ± 7.2###	89.8 ± 15.4*	90.7 ± 6.3***
JIP3 (ng/L)	233.9 ± 41.7	379 ± 35.9##	274.7 ± 57	219.9 ± 40.9**
p-JNK/JNK	5.3 ± 0.6	8.4 ± 1.0##	5.3 ± 0.8*	5.4 ± 0.6*
p-APP/APP	0.2 ± 0.0	0.4 ± 0.1##	0.2 ± 0.1**	0.3 ± 0.1**
Aβ (mg/L)	2.5 ± 0.2	3.0 ± 0.1#	2.4 ± 0.2**	2.3 ± 0.2**
TNF-α (ng/L)	132.6 ± 13.2	189.7 ± 22.6#	101.5 ± 25.3**	103 ± 17.7**
IL-1β (ng/L)	91 ± 6.1	124.9 ± 8.9#	95.9 ± 4.9**	87.8 ± 7.6**
IL-6 (pg/mL)	193.1 ± 8.9	223.7 ± 7.8#	190.2 ± 7.5**	177.9 ± 11.9**
IL-8 (pg/mL)	367.3 ± 23.2	471.8 ± 22.9##	364.1 ± 37.8*	310.3 ± 22.9***
IL-12 (ng/L)	58.6 ± 6.5	82.3 ± 4.0##	69.5 ± 3.5*	67.2 ± 2.9*

Data are expressed as the mean ± standard error (*n* = 8)

^#^*p* < 0.05

^##^*p* < 0.01

^###^*p* < 0.001 vs. WT mice

*p < 0.05

**p < 0.01

***p < 0.001 vs. APP/PS1 mice

**Table 2:** The effects of FTS•B on cytokines levels in the brains of APP/PS1 mice

	WT	APP/PS1		
			FTS•B (mg/kg)	
			10	40
TLR3 (ug/g prot)	30.7 ± 2.9	22.7 ± 1.9##	27.6 ± 2.8*	28.3 ± 2.3*
p-IRF3/IRF3	0.2 ± 0.0	0.2 ± 0.0#	0.5 ± 0.1*	0.5 ± 0.1***
IFN-β (ng/g prot)	30.7 ± 3.7	17.3 ± 2.5#	23.4 ± 4.1	26.4 ± 1.9*
JIP3 (ng/g prot)	81.4 ± 17	171.8 ± 35.2#	59.6 ± 21.3*	53.8 ± 10.6**
p-JNK/JNK	7.1 ± 0.6	10 ± 1.0#	5.6 ± 1.1*	5.7 ± 0.7**
p-APP/APP	0.3 ± 0.0	0.6 ± 0.1#	0.2 ± 0.1**	0.2 ± 0.0**
Aβ (mg/g prot)	1.1 ± 0.1	1.5 ± 0.1##	0.9 ± 0.1***	1.2 ± 0.1**
TNF-α (ng/g prot)	2.1 ± 0.1	2.3 ± 0.1#	1.7 ± 0.1***	1.8 ± 0.1**
IL-1β (ng/g prot)	68.4 ± 8.1	113.6 ± 13.1##	82.5 ± 10.8*	75.4 ± 6.4**
IL-6 (ng/g prot)	176.7 ± 4.3	215.7 ± 8.3###	171.2 ± 8.4***	170.4 ± 9.8**
IL-8 (ng/g prot)	96 ± 15.1	177.5 ± 28.1#	96.2 ± 18.5*	105.5 ± 9.1
IL-12 (ng/g prot)	37.6 ± 3.0	56 ± 5.3#	29.7 ± 5.6**	29 ± 4.2**

Data are expressed as the mean ± standard error (*n* = 8)

^#^*p* < 0.05

^##^*p* < 0.01

^###^*p* < 0.001 vs. WT mice

*p < 0.05

**p < 0.01

***p < 0.001 vs. APP/PS1 mice

### FTS•B-mediated regulation of neuroinflammation-related protein expression and activation in the hippocampus of APP/PS1 mice

**Fig. 4:**
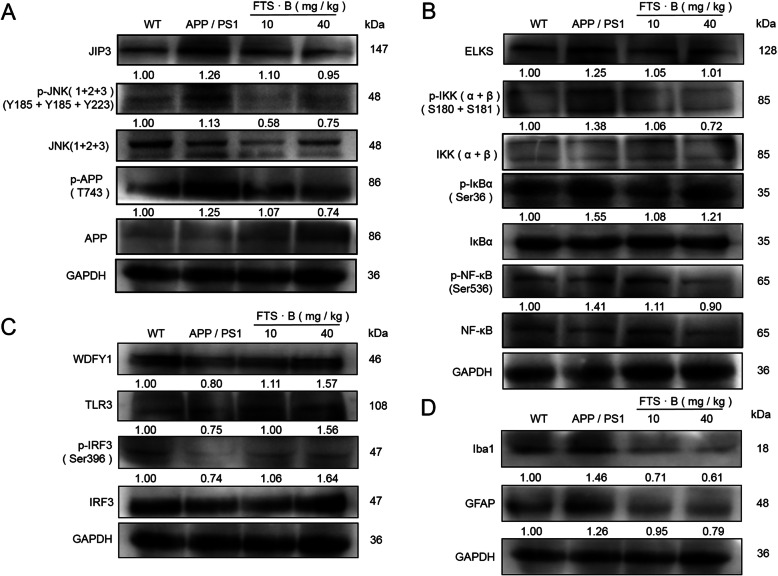
The regulation of FTS•B on proteins related to neuroinflammation in the hippocampus of APP/PS1 mice. (**a**) FTS•B reduced the expression levels of JIP3 and the phosphorylation levels of JNK and APP. (**b**) FTS•B suppressed the expression levels of ELKS and the phosphorylation levels of IKK(α + β), IκBα, and NF-κB p65. (**c**) FTS•B enhanced the expression levels of WDFY1 and TLR3, as well as the phosphorylation levels of IRF3. (**d**) FTS•B reduced the expression levels of Iba1 and GFAP. Quantification data were normalized to GAPDH and corresponding total proteins (*n* = 3). They were reported as folds of the corresponding WT group. FTS•B, forsythoside B; APP/PS1, amyloid precursor protein/presenilin 1; JIP3, JNK-interacting protein 3; JNK, C-Jun NH2-terminal kinase; APP, amyloid-beta precursor protein; IKK, inhibitor of nuclear factor kappa-B kinase; IκBα, inhibitor of nuclear factor kappa-B alpha; NF-κB, nuclear factor-κB; WDFY1, WD-repeat and FYVE-domain-containing protein 1; TLR3, toll-like receptor 3; IRF3, interferon regulating factor 3; Iba1, ionized calcium-binding adapter molecule 1; GFAP, glial fibrillary acidic protein

To further study the mechanism of FTS•B-mediated anti-neuroinflammation, the activation or phosphorylation of JIP3, JNK, APP, ELKS, IKKα+IKKβ, IκBα, NF-κB, WDFY1, TLR3, IRF3, Iba1, and GFAP in the hippocampus were assessed using western blotting. Compared with the vehicle-treated APP/PS1 mice, FTS•B visibly downregulated the expression of JIP3, p-JNK(1 + 2 + 3), and p-APP in the hippocampus (Fig. 4a). ELKS can combine with the IKK complex and is involved in the activation of NF-κB [38]. FTS•B administration suppressed the levels of ELKS, p-IKK(α + β), p-IκBα, and p-NF-κB (Ser536) in the hippocampus of APP/PS1 mice (Fig. 4b), while enhancing the levels of WDFY1, TLR3, and p-IRF3 (Fig. 4c). Furthermore, compared with the vehicle-treated APP/PS1 mice, FTS•B downregulated Iba1 by 51.4% and GFAP by 24.6% in the hippocampus (Fig. 4d).

### FTS•B-mediated prevention of LPS-induced neuroinflammation in BV-2 cells

**Fig. 5:**
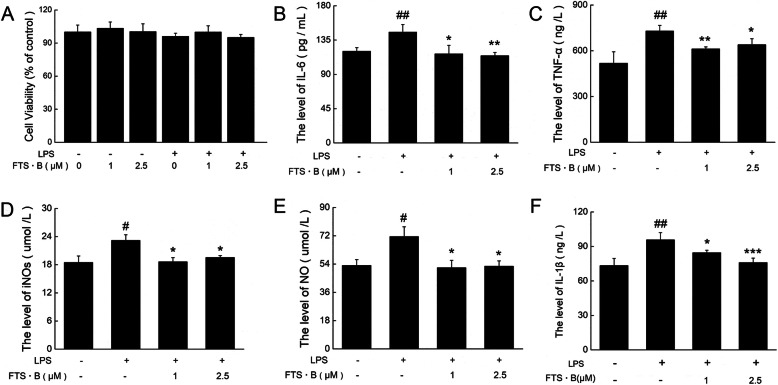
FTS•B incubation regulated the levels of inflammatory cytokines in LPS-exposed BV-2 cells. (**a**) FTS•B alone or in co-incubation with LPS showed no effects on cell viability of BV-2 cells. Compared with LPS-alone exposed cells, FTS•B co-incubation strongly suppressed the release of (**b**) IL-6, (**c**) TNF-α, (**d**) iNOs, (**e**) NO, and (**f**) IL-1β in BV-2 cells, determined by ELISA. Data are expressed as mean ± standard error (*n* = 3). #*p* < 0.05 and ##*p* < 0.01 vs. non-treated cells. *p < 0.05, **p < 0.01, and ***p < 0.001 vs. LPS-alone exposed cells. FTS•B, forsythoside B; LPS, lipopolysaccharide; IL-6, interleukin 6; TNF-α, tumor necrosis factor α; iNOs, inducible nitric oxide synthase; NO, nitric oxide; IL-1β, interleukin 1β; ELISA, enzyme-linked immunosorbent assay

FTS•B alone (1 μM and 2.5 μM) or in co-incubation with LPS (1 μg/mL) showed no significant effects on the cell viability of BV-2 cells (Fig. 5a). The LPS-induced enhancements of IL-6 (*p* < 0.01; Fig. 5b), TNF-α (*p* < 0.01; Fig. 5c), iNOs (*p* < 0.05; Fig. 5d), NO (*p* < 0.05; Fig. 5e), and IL-1β (*p* < 0.01; Fig. 5f) in BV-2 cells were strongly suppressed by FTS•B pre-incubation (*p* < 0.05; Fig. 5b-f).

### FTS•B-mediated reduction in microglia-mediated neurotoxicity

**Fig. 6:**
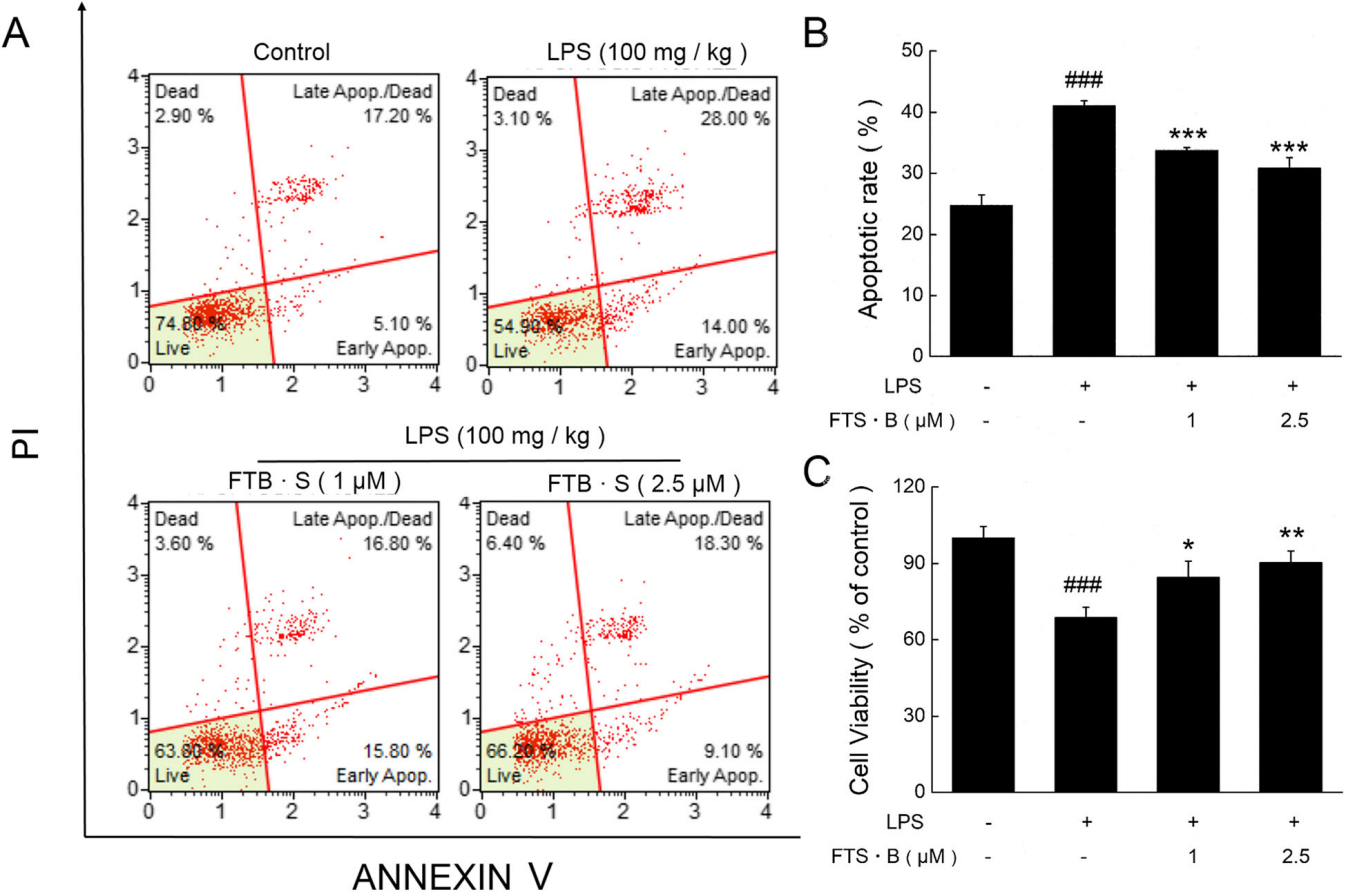
FTS•B reduced the microglia-mediated neurotoxicity induced by LPS. Mouse HT22 cells were treated using conditioned media from BV-2 microglia exposed to LPS for 24 h, with or without FTS•B. (**a** and **b**) The apoptosis rate and (**c**) cell viability were assessed. Data are expressed as mean ± standard error (*n* = 3). ###*p* < 0.001 vs. control group. *p < 0.05, **p < 0.01, and ***p < 0.001 vs. group treated with LPS. FTS•B, forsythoside B; LPS, lipopolysaccharide

Activated microglia exert neurotoxic effects by releasing pro-inflammatory enzymes and mediators [39]. When conditioned media from LPS-stimulated microglia were added to cultured HT22 cells, the apoptosis rate of these cells was significantly increased (*p* < 0.001; Fig. 6a and b), and cell viability was significantly reduced (*p* < 0.001; Fig. 6c). However, pretreatment of BV-2 cells with FTS•B prior to LPS stimulation significantly suppressed the apoptosis rate of the HT22 cells (*p* < 0.001; Fig. 6a and b) and enhanced cell viability (*p* < 0.05; Fig. 6c), demonstrating the neuroprotective effects of FTS•B.

## Discussion

In the present study, we found FTS•B attenuates AD symptoms, pathology, and decreases neuroinflammation signaling and cytokines in mice models and cell culture. These findings, for the first time, prove FTS•B modulate neuroinflammation in the central nervous system and inhibit the progression of AD, suggesting FTS•B is a potential therapeutic medicine in the future. These findings also support the hypothesis that neuroinflammation mediates the progression of AD [40].

4-HNE, Aβ, and neurofibrillary are the central pathological mechanism of AD. 4-HNE triggers Aβ aggregation by accelerating Aβ protofibril formation [41]. Tauopathy in the form of neurotic plaques and cerebral β-amyloidosis in the form of Aβ plaques are two possible causes of the pathological features of AD [42]. Neurofibrillary tangles consist of intracellular bundles of self-assembled p-tau, which are induced by Aβ deposition and lead to neuronal degeneration [43]. APP/PS1 double-transgenic mice with AD imitate human progressive cognitive deficits and neuropathological characteristics, including neuroinflammation [44]. By using APP/PS1 AD mice, we showed FTS•B significantly enhanced the learning and memory capabilities of APP/PS1 mice and markedly suppressed the deposition of Aβ, the formation of neurofibrillary tangles composed of p-tau protein, and the levels of 4-HNE in the hippocampus. These results suggest FTS•B administration decreases the Aβ accumulation and its downstream pathological effects to protect neuronal damages in AD mice.

Neuroinflammation is associated with AD and is hypothesized to be the key mediator of AD [40]. Neuroinflammation is characterized by Aβ activated microglia which secretes neurotoxic mediators and pro-inflammatory cytokines to activate astrocytes to be neurotoxic [45, 46]. Neuroinflammation causes neuronal death by neurotoxic astrocytes which upregulates complement cascade and neurotoxins [47, 48]. Further, neuroinflammation can amplify itself by increasing tauopathy and Aβ deposition through microglia-secreted pro-inflammatory cytokines such as IL-6 and TNF-α [49]. A key mediator of neuroinflammation is the activation of NF-κB in astrocyte and microglial cells. Latent NF-κB can be activated by many inflammatory stimulations such as Aβ [8], transport-activated JNK3 [50], neurofibrillary tangles [51], and TNF-α [52]. Activated NF-κB increases the production of IL-1β, TNF-α, iNOS, and IL-6 [53, 54]. Another key mediator of neuroinflammation is JNK in the central nervous system. Patients with AD show upregulated expression of JNK in the brains, which promotes amyloidogenic APP cleavage and amyloid plaque formation [55]. Activated astrocytes and microglia increases the expression of GFAP and Iba1, respectively [33], and we showed FTS•B reduced the expression of GFAP and Iba1 in the hippocampus, indicating that FTS•B suppressed the activation of astrocytes and microglia in AD mice. We showed FTS•B decreases the NF-κB and JNK inflammatory pathways and inflammatory cytokines including IL-6, TNF-α, IL-1β, IL-8, and IL-12, suggesting FTS•B inhibits the inflammatory signaling pathways in AD mice. WDFY1 overexpression enhances TLR3-mediated NF-κB activation and IRF3 phosphorylation, inducing the production of IFNs and various pro-inflammatory cytokines [56]. Interestingly, we showed FTS•B increases WDFY1 expression, activates TLR3-IRF3 pathways, and increases IFN-β production, suggesting FTS•B may improve the neuronal function and autophagy [57]. These results suggest FTS•B suppresses the activation of microglia and astrocytes and neuroinflammation, while maintaining proper neuronal function.

In vivo experiment did not prove the downregulation of inflammatory cytokines is due to the inactivation of microglia. Thus, we use tissue culture of microglia cells as a model. BV-2 microglial cells is an effective substitute for primary microglia [58]. When induced by LPS, it shows properties associated with inflammation, which imitates the neuroinflammation in AD mice and AD patient individuals [59]. We used LPS-induced BV-2 microglial activation as a classic inflammatory model to evaluate the anti-neuroinflammatory effects of potential therapeutic candidates [60]. In the present study, FTS•B significantly suppressed the production of pro-inflammation cytokines in BV-2 cells stimulated by LPS. It was reported that the activated microglia develops neurotoxicity effects via the release of pro-inflammatory mediators [61]. The co-culture system of neuron-microglia provides an environment for them to grow together, which is diffusely used to detect the neuroprotective effects of anti-inflammatory drugs [62]. Hence, hippocampal HT22 cells were used as a neuronal model to confirm the protective effect of FTS•B on LPS-induced BV-2 microglia toxicity. The conditioned media from LPS-induced BV-2 cells was diffused into the HT-22 neuronal cells, leading to the HT-22 apoptosis. FTS•B suppressed the apoptosis rate of HT22 cells exposed to medium containing the secondary metabolite of BV-2 cells stimulated by LPS. These results suggest FTS•B increases neuronal survival through suppressing the activation of microglia.

Current treatment of AD involves N-methyl-D-aspartate receptor antagonists, acetylcholinesterase inhibitors, and non-steroidal anti-inflammatory drugs. These drugs have disadvantages: inefficiency, tolerance, and side-effects. We perform acute toxicity trials and histopathological examinations of the organs of APP/PS1 mice to show that FTS•B is safe to use, with no obvious side effects. In the entire experiment of 36 days, FTS•B completely attenuated the AD symptoms and pathological features. These results suggest FTS•B is a new and safe candidate drug for AD treatment in the future.

The present study had some limitations. First, FTS•B is a natural product that targets multiple cell types and molecules which means FTS•B may target molecules other than inflammation. Although FTS•B supports the hypothesis that neuroinflammation might mediate the progression of AD, we need further mechanism studies to illustrate whether neuroinflammation is the major mediator of AD. For example, we may restore NF-κB activity and see whether Aβ deposition is restored. Second, we did not measure how much FTS•B passing through the blood-brain barrier, so we do not know whether FTS•B directly targets brain cells or reduces systemic inflammation and indirectly attenuate neuronal inflammation. A potential solution to this issue may be the use of a NF-κB reporter mice treated with FTS•B.

Our proteomics and western blot studies show upregulated proteins (TLR3, p-IRF3, IFN-β, and WDFY1) and downregulated proteins (JIP3, p-JNK, p-APP, ELKS, p-IKK, p-IκBα, and p-NFκB). These proteins suggest FTS•B modifies NF-κB pathways. In the future, we will identify the downstream target of FTS•B among these candidate proteins and study the molecular mechanisms of FTS•B to illustrate how FTS•B protects from AD at the molecular level.

## Conclusions

In summary, the present study was the first to systematically report that FTS•B exhibited neuroprotective effects in APP/PS1 mice, ameliorated Aβ deposition and tau protein phosphorylation, and attenuated microglia and astrocyte activation, thus restoring cognitive function. FTS•B prevented LPS-induced neuroinflammation and reduced microglia-mediated neurotoxicity in BV-2 and HT22 cells. The neuroprotective effect of FTS•B may be mediated by its anti-neuroinflammatory activities, which are regulated via the NF-κB signaling pathway. The results suggest that FTS•B shows safe and promising therapeutic effects in AD treatment. The results also support the hypothesis that neuroinflammation is a key mediator of AD [40]. Further mechanism study is needed to illustrate the molecular mechanism of FTS•B and the contribution of neuroinflammation in the progression of AD.

## Supplementary information

**Additional file 1: Table S1.** The body weights of WT mice and APP/PS1 mice. **Table S2.** Details of the significantly up- or down-regulated proteins by FTS•B among experimental groups. **Figure S1.** Chemical structure of FTS•B. **Figure S2.** The process of the drug administration and behavioral tests. **Figure S3.** Compared with normal saline group, 400 mg/kg of FTS•B treatment caused no substantial changes in liver, spleen, kidney and brain tissues. (A) Liver, (B) spleen, (C) kidney and (D) brain were detected by H&E staining (200×; scale bar = 50μm). No substantial changes were noted among all experimental mice. FTS•B, forsythoside B; H&E, hematoxylin and eosin. **Figure S4.** FTS•B caused no substantial changes in liver, spleen, kidney and brain tissues. (A) Liver, (B) spleen, (C) kidney and (D) brain were detected by H&E staining (200×; scale bar = 50 μm). No substantial changes were noted among all experimental mice. FTS•B, forsythoside B; H&E, hematoxylin and eosin.

## Data Availability

The datasets used and/or analyzed during the current study are available from the corresponding author on reasonable request.

## Abbreviations

MTT: 3-(4, 5-dimethyl-2-thiazolyl) 2, 5-diphenyl-2H-tetrazolium bromide assay
4-HNE: 4-hydroxynonenal
ACN: Acetonitrile
BCA: Bicinchoninic acid
AD: Alzheimer’s disease
Aβ: Amyloid-beta
APP: Amyloid-beta precursor protein
APP/PS1: Amyloid precursor protein/presenilin 1
JNK: C-Jun NH2-terminal kinase
DMEM: Dulbecco’s modified Eagle’s medium
ELISA: Enzyme-linked immunosorbent assay
FA: Formic acid
FTS•B: Forsythoside B
GFAP: Glial fibrillary acidic protein
H&E: Hematoxylin and eosin
HRP: Horseradish peroxidase
IκBα: Inhibitor of nuclear factor kappa-B alpha
iNO_S_: Inducible nitric oxide synthase
IKKα: Inhibitor of nuclear factor kappa-B kinase alpha
IKKβ: Inhibitor of nuclear factor kappa-B kinase beta
IFN-β: Interferon-beta
IRF3: Interferon regulating factor 3
IL-1β: Interleukin-1 beta
IL-6: Interleukin-6
IL-8: Interleukin-8
IL-12: Interleukin-12
Iba1: Ionized calcium-binding adapter molecule 1
JIP3: JNK-interacting protein 3
LPS: Lipopolysaccharide
NO: Nitric oxide
NF-κB: Nuclear factor-κB
PMSF: Phenylmethanesulfonyl fluoride
PBS: Phosphate buffered saline
RIPA: Radio immunoprecipitation assay
GAPDH: Reference protein glyceraldehyde-3-phosphate dehydrogenase
TLR3: Toll-like receptor 3
TNF-α: Tumor necrosis factor-alpha
WDFY1: WD-repeat and FYVE-domain-containing protein 1
