# Supplementary material for: Forsythoside B attenuates memory impairment and neuroinflammation via inhibition on NF-κB signaling in Alzheimer’s disease
Source: J Neuroinflammation. 2020 Oct 15;17:305. doi: 10.1186/s12974-020-01967-2 (PMC7565774; doi:10.1186/s12974-020-01967-2)
Supplement: Supplementary file 1 — Additional file 1: Table S1. The body weights of WT mice and APP/PS1 mice. Table S2. Details of the significantly up- or down-regulated proteins by FTS•B among experimental groups. Figure S1. Chemical structure of FTS•B. Figure S2. The process of the drug administration and behavioral tests. Figure S3. Compared with normal saline group, 400 mg/kg of FTS•B treatment caused no substantial changes in liver, spleen, kidney and brain tissues. (A) Liver, (B) spleen, (C) kidney and (D) brain were detected by H&E staining (200×; scale bar = 50μm). No substantial changes were noted among all experimental mice. FTS•B, forsythoside B; H&E, hematoxylin and eosin. Figure S4. FTS•B caused no substantial changes in liver, spleen, kidney and brain tissues. (A) Liver, (B) spleen, (C) kidney and (D) brain were detected by H&E staining (200×; scale bar = 50 μm). No substantial changes were noted among all experimental mice. FTS•B, forsythoside B; H&E, hematoxylin and eosin. [file 12974_2020_1967_MOESM1_ESM.docx]

Table S1. The body weights of WT mice and APP/PS1 mice.

| Days | WT | APP/PS1 | | |
| --- | --- | --- | --- | --- |
|  |  |  | FTS•B (mg/kg) | |
|  |  |  | 10 | 40 |
| 1^st^ | 42.9±0.9 | 43.6±0.8 | 43.7±0.5 | 42.6±1.3 |
| 7^th^ | 43.4±1.2 | 43.3±1.1 | 44.1±1.5 | 41.2±1.2 |
| 14^th^ | 42.9±1.1 | 43.7±0.7 | 43.7±1.3 | 41.7±1.5 |
| 21^st^ | 42.2±1 | 43.1±1.3 | 44.2±1.5 | 41.7±1.5 |
| 28^th^ | 43±2.1 | 43.5±1.3 | 44.1±2.2 | 43.4±0.4 |

Data are expressed as the mean ± standard error (n=8).

Table S2. Details of the significantly up- or down-regulated proteins by FTS•B among experimental groups.

| Symbol | Unique.  peptides | fc..APP/PS1  -WT | fc.. FTS•B  - APP/PS1 | Symbol | Unique.  peptides | fc..APP/PS1  -WT | fc.. FTS•B- APP/PS1 | |
| --- | --- | --- | --- | --- | --- | --- | --- | --- |
| Upregulated proteins by FTS•B (Number: 16) | | | | | | | |  |
| Tmsb4x | 4 | 0.01 | 235.26 | S100a1 | 4 | 0.40 | 2.47 | |
| Pfdn1 | 7 | 0.05 | 20.74 | Wdfy1 | 6 | 0.41 | 2.43 | |
| Actc1 | 3 | 0.10 | 12.26 | Stk32c | 4 | 0.60 | 1.14 | |
| Praf2 | 2 | 0.14 | 8.26 | Vti1b | 5 | 0.76 | 1.24 | |
| Ech1 | 3 | 0.20 | 4.79 | Slc39a10 | 4 | 0.76 | 1.27 | |
| Kcnj10 | 5 | 0.29 | 1.92 | Ampd2 | 7 | 0.84 | 1.17 | |
| Ddt | 4 | 0.36 | 2.77 | mCG_6739 | 2 | 0.93 | 1.12 | |
| Baiap3 | 2 | 0.38 | 3.17 | Jam3 | 4 | 0.96 | 1.22 | |
| Downregulated proteins by FTS•B (Number: 7) | | | | | | | | |
| C1qb | 3 | 1.46 | 0.70 | Mapk8ip3 | 4 | 5.07 | 0.29 | |
| Gria3 | 2 | 1.70 | 0.14 | Metap2 | 3 | 5.36 | 0.28 | |
| Erc1 | 4 | 1.86 | 0.27 | Arpp21 | 3 | 6.63 | 0.09 | |
| Safb | 3 | 4.18 | 0.17 |  |  |  |  | |

**
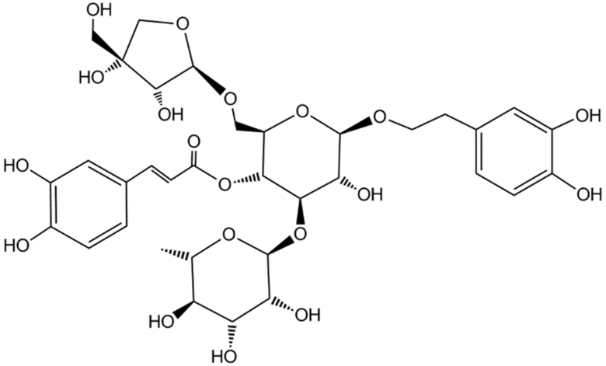
**

Figure S1. Chemical structure of FTS•B.


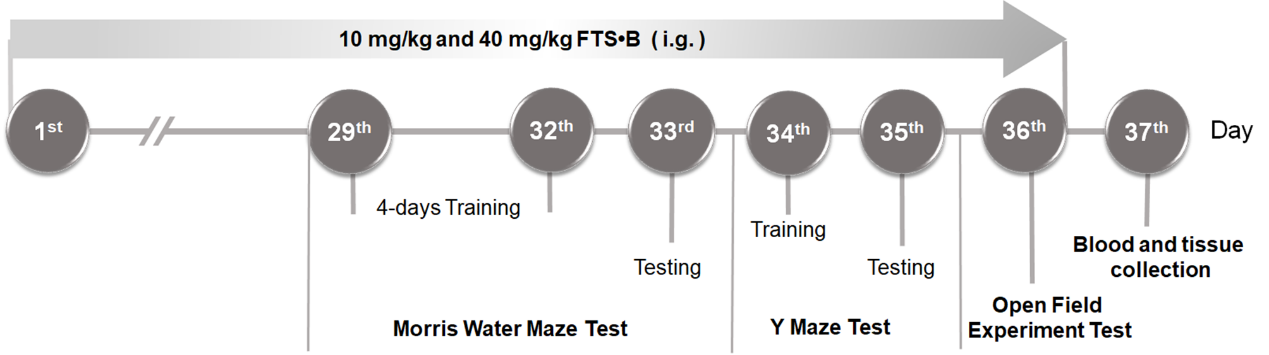


Figure S2. The process of the drug administration and behavioral tests.


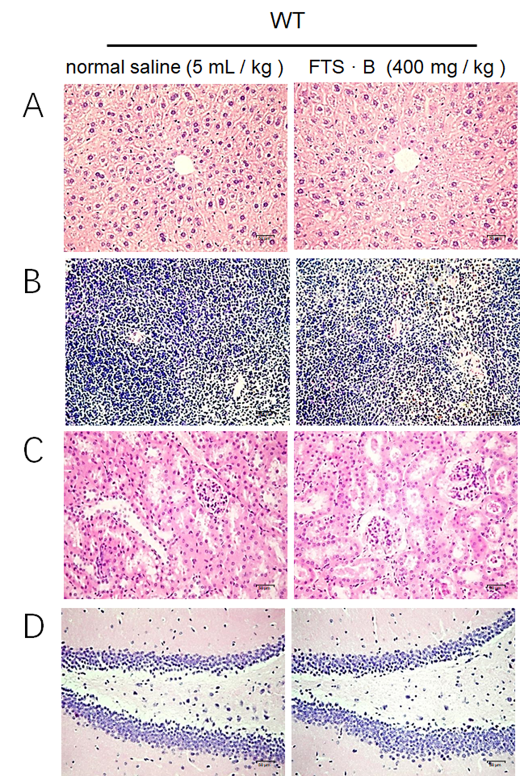


Figure S3. Compared with normal saline group, 400 mg/kg of FTS•B treatment caused no substantial changes in liver, spleen, kidney and brain tissues. (A) Liver, (B) spleen, (C) kidney and (D) brain were detected by H&E staining (200×; scale bar = 50 μm). No substantial changes were noted among all experimental mice.

FTS•B, forsythoside B; H&E, hematoxylin and eosin.

**
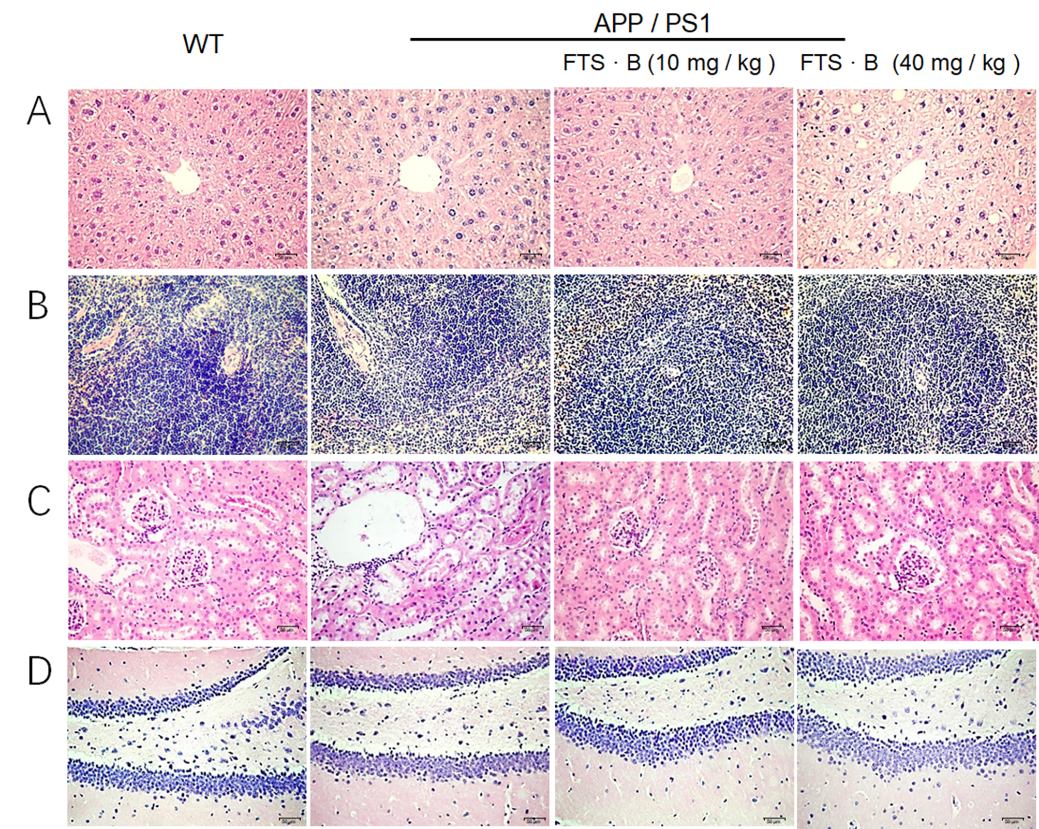
**

Figure S4. FTS•B caused no substantial changes in liver, spleen, kidney and brain tissues. (A) Liver, (B) spleen, (C) kidney and (D) brain were detected by H&E staining (200×; scale bar = 50 μm). No substantial changes were noted among all experimental mice.

FTS•B, forsythoside B; H&E, hematoxylin and eosin.
